# Antigen-Mimic Nanoparticles in Ultrasensitive on-Chip Integrated Anti-p53 Antibody Quantification

**DOI:** 10.1021/acssensors.3c02568

**Published:** 2024-03-05

**Authors:** Shaoyu Kang, Daohe Yuan, Robert Barber, Jason J. Davis

**Affiliations:** Department of Chemistry, University of Oxford, South Parks Road, Oxford OX1 3QZ, U.K.

**Keywords:** nanoparticle-assisted immunoisolation, electrochemical enzyme-amplified assay, p53, antigen-mimicking peptide, cancer detection

## Abstract

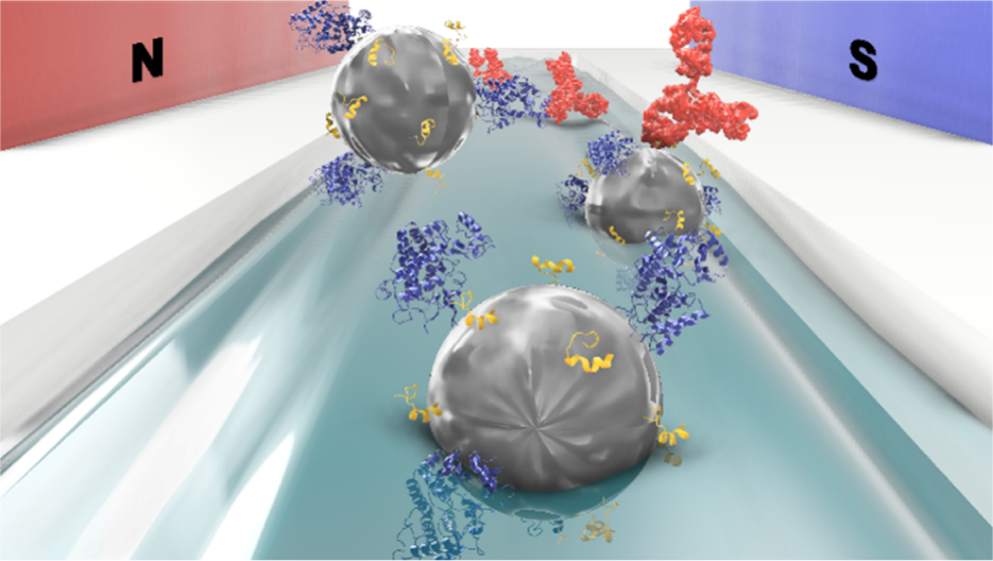

As a tumor-suppressing protein, p53 plays a crucial role in preventing cancer development. Its utility as an early cancer detection tool is significant, potentially enabling clinicians to forestall disease advancement and improve patient prognosis. In response to the pathological overexpression of this antigen in tumors, the prevalence of anti-p53 antibodies increases in serum, in a manner quantitatively indicative of cancer progression. This spike can be detected through techniques, such as Western blotting, immunohistochemistry, and immunoprecipitation. In this study, we present an electrochemical approach that supports ultrasensitive and highly selective anti-p53 autoantibody quantification without the use of an immuno-modified electrode. We specifically employ antigen-mimicking and antibody-capturing peptide-coated magnetic nanoparticles, along with an AC magnetic field-promoted sample mixing, prior to the presentation of Fab-captured targets to simple lectin-modified sensors. The subfemtomolar assays are highly selective and support quantification from serum-spiked samples within minutes.

Cancer is defined as the uncontrolled proliferation and spread of abnormal cells, culminating in tumor formation, and the subsequent invasion of adjacent tissues and organs.^1^ As a major contributor to global mortality, it accounted for approximately one-sixth of all deaths in 2020, with some 10 million fatalities.^2^ This figure is estimated to reach 27 million per annum over the coming decade.^3^ Against this backdrop, it is clear that early detection is critical to the improved patient outcome. Among the myriad of cancer biomarkers, p53, encoded by the TP53 gene, has gained prominence due to its core antiproliferative function in preserving genomic stability.^4^ In more than 50% of human cancers,^5^ aberrant p53 proteins, encoded by a mutated TP53, accumulate in cancer cells and may further promote tumor growth and metastasis.^1,5,6^ This accumulation manifests as an increased concentration of p53 proteins in serum and has, for example, been assayed at levels >300% higher than those of healthy controls in patients with gastrointestinal cancer^7^ and >200% higher in lung cancer.^8^ The robust assaying of circulating p53 is, however, made challenging due to both the heterogeneity of both its mutated forms and post-translational modifications.^7,9^ The abnormal accumulation of p53 proteins triggers the generation of anti-p53 antibodies.^10^ These antibodies are largely structurally consistent, and their quantification, at levels (∼100 ng/mL), i.e., spiking to hundreds of times higher than that of the antigen in serum,^11^ is more accessible. Their assaying could provide a potentially more robust and direct insight into cancer progression and prognosis.^12^ Among the anti-p53 antibodies, the monoclonal DO-1 antibody is widely used in Western blotting, immunohistochemistry, and immunoprecipitation.^5^ It is known to bind to a relatively conserved six-residue epitope (SDLWKL) on the N-terminal transactivation domain (TAD) of p53.^12^ This region has been shown to be less prone to mutation compared to the DNA-binding domain (DBD), making it a more consistently effective target for antibodies regardless of p53 form.^13^

A broad range of routes to anti-p53 antibody quantification, of course, exist.^5^ Of these, electrochemical sensors are unique in terms of cost-effectiveness, scalability, and analytical performance.^14^ Typically, these assays utilize electrode-confined p53 antigens.^15^ However, on planar two-dimensional interfaces, the combined effects of moderately low epitope surface density (∼pmol/cm^2^),^16^ restricted target accessibility, and sluggish (planar) target diffusion serve to reduce the efficacy of large target (e.g., antibody) capture. Additionally, in protein-rich real samples (e.g.*,* serum), recruitment against a large excess of background remains challenging. Recently, peptide-based receptors have emerged as a promising alternative to immunoprotein counterparts, finding utility across a broad spectrum of applications spanning diagnostics and therapeutics.^17−20^ These versatile recognition elements can exhibit a high binding specificity and affinity (nM *K*_d_), good chemical stability, tunability, and substantially reduced cost.^17,18,21^ Herein, we have utilized solution-phase peptide-presenting magnetic nanoparticles, with sequences derived from the known p53 epitope (SDLWKL) to selectively recruit serum-based anti-p53 antibodies.

Nanomaterials have been progressively incorporated into sensor formats, offering rich interfacial functionality and greater biological loading relative to planar surfaces.^22^ When free in solution, their associated three-dimensional (3D) target recruitment is particularly advantageous,^23,24^ as exemplified across a broad range of modified iron oxide nanoparticles (IONPs).^24,25^ Herein, we specifically utilize dual-modified IONPs (2.1 kDa 18-amino acid peptide recognition element and horseradish peroxidase, HRP) to recruit antibodies from serum prior to presenting them to lectin-modified screen-printed sensors (Figure 1). Concanavalin A (Con A) has a high affinity for mannose and glucose residues,^26^ a characteristic that has been leveraged to bind antibodies (e.g., immunoglobulin G)^27^ to planar surfaces. An alternating current (AC) magnetic field (MAC) was used to promote solution-phase target capture in a 3D-printed microfluidic format, with the antibody–IONP complexes subsequently transferred to a Con A-modified electrode. Here, the HRP-mediated catalytic oxidation of a TMB substrate generates a voltammetric response that reports directly and quantitatively on the anti-p53 concentration. The employed antigenic peptide exhibits high sequence specificity, with a target recruitment performance superior to that associated with the full antigen under equivalent conditions. The enzyme-amplified downstream assay is exceptionally sensitive and selective in serum-spiked samples, supporting potential for clinical translation.

**Figure 1:**
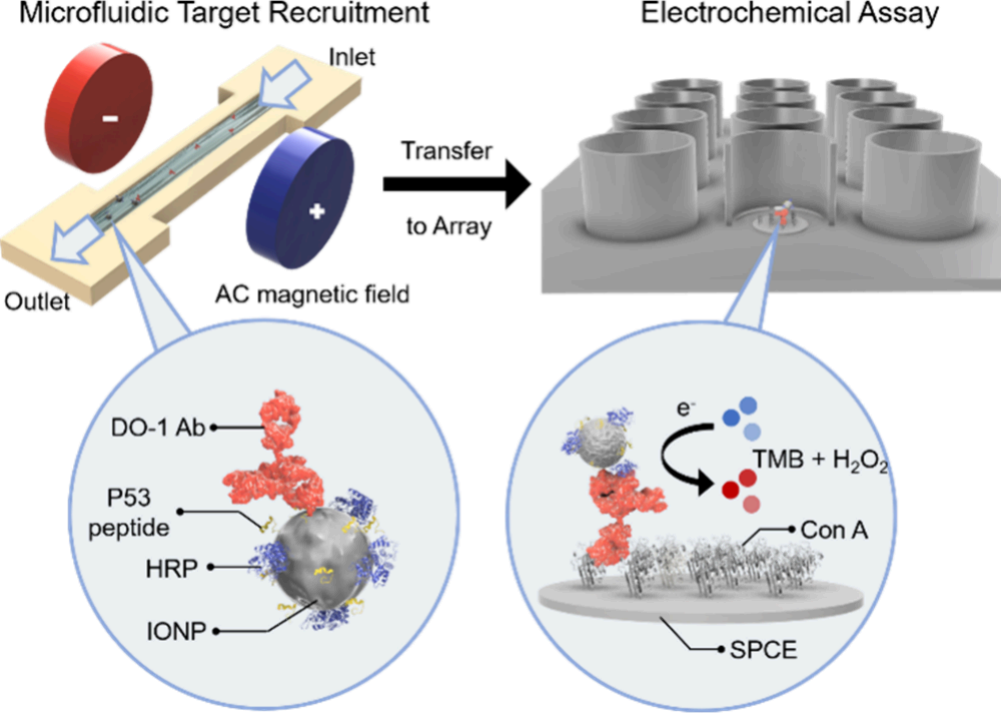
Schematic depiction of the nanoparticle-assisted immunocapture and downstream electrochemical enzyme-amplified assay for the DO-1 antibody quantification. The synthesized antigen-mimic was covalently tethered to IONP surfaces, along with HRP (bottom left). An AC magnetic field was employed to enhance mixing of the capture IONPs with 100 μL samples within a microfluidic channel (upper left). After immunocapture, the target–nanoparticle complexes were manually transferred to 200 μL microplate wells, encompassing Con A-modified screen-printed carbon electrode (SPCE) interfaces (upper right). In the presence of the tetramethylbenzidine substrate (TMB) and H_2_O_2_, a concentration-dependent SWV response is then generated (bottom right).

## Experimental Section

### Materials and Instruments

Detailed experimental protocols, including the materials and instruments used throughout, are provided in the Supporting Information. All SPR analyses were conducted at room temperature (298 K) and at a flow rate of 30 μL/min in 10 mM PBS running buffer (pH = 7.4), unless otherwise specified. Detailed SPR protocols are provided in the Supporting Information. All electrochemical experiments were conducted using a PalmSens4 potentiostat (PalmSens BV) on screen-printed carbon electrode (SPCE) arrays (FlexMedical Solutions). These arrays comprised a carbon working electrode (3.0 mm diameter), a carbon counter electrode, and a silver/silver chloride (Ag/AgCl) reference electrode. Prior to use, SPCE arrays were cleaned by washing three times with ethanol and water, followed by a 30 min plasma cleaning under an atmosphere of 0.1 atm O_2_.

### Bioreceptive IONP Synthesis

Low-polydispersity IONPs were prepared hydrothermally.^28^ Briefly, a solution was prepared by sonication and stirring of a mixture of ethylene glycol (50 mL), anhydrous trisodium citrate (0.65 g), anhydrous iron(III) chloride (0.97 g), and anhydrous sodium acetate (3.00 g) at room temperature for 30 min. Subsequently, water (0.25 mL) was introduced. The homogenized yellow mix was transferred into an autoclave and heated at 200 °C for 10 h and then allowed to cool down to room temperature overnight. The resulting particles were purified by washing three times with water and ethanol, collected using a Neodymium disc magnet, and then dried overnight at 60 °C.

Bioreceptive nanoparticles were prepared using active ester EDC-NHS coupling chemistry. Initially, the particles were washed three times with a 15 mM MES buffer solution (pH = 5.5) and then dispersed into a 0.2 M EDC–0.05 M NHS MES solution (pH = 5.5) and incubated on a roller for 1 h at room temperature. The activated particles were then suspended in a solution containing 50 μg/mL HRP and 5 μg/mL p53 peptide in MES (pH = 5.5), followed by overnight incubation on a roller at room temperature. After a PBS wash (pH = 7.4), particle surfaces were blocked with 1 mg/mL BSA in a PBS-T solution and then washed with PBS.

### Nanoparticle-Assisted Immunoisolation

A microfluidic platform to facilitate magnetic mixing and isolation of the IONP/Ab conjugates was prepared by 3D printing (see Figure S1 for details). The sample and running buffer were delivered into the microfluidic channel at a controlled flow rate (25 to 200 μL·min^–1^) by an automated syringe pump. A pair of opposing 150 N electromagnets (RS Components Ltd., UK) were mounted to the microfluidic configuration. These were connected to an external, programmable power supply generating an alternating electromagnetic field at a (potential) adjustable field strength. To initiate the assay, a 100 μL aliquot of peptide-modified IONPs (10 mg/mL) was introduced into the microfluidic channel. Under an alternating electromagnetic field, the target DO-1 antibody, at varying concentrations, was then injected at a fixed flow rate of 50 μL·min^–1^. After 10 min of immunoisolation under optimized microfluidic conditions (see below), the resulting antibody–IONP complexes were eluted from the channel and manually transferred by pipet onto Con A-modified SPCEs (prepared by overnight incubation of 100 μg/mL Con A) for electrochemical assaying.

### Electrochemical Enzyme-Amplified Assay

Following a 10 min incubation of the antibody–IONP immunocomplexes on Con A-modified SPCEs, and subsequent washing with PBS, 100 μL of the high-sensitivity TMB substrate was added and left to react for 5 min. Square-wave voltammetry (SWV) scans were then conducted from +1.0 to −0.5 V (vs Ag/AgCl), at an amplitude of 20 mV, a potential step of 2 mV, and a frequency of 50 Hz. Specificity tests were carried out with 1.0 mg/mL human serum albumin (HSA), bovine serum albumin (BSA), 1.0% human serum, and 10 μg/mL IgG prepared in a PBS buffer. Recovery assays were conducted using DO-1 antibodies spiked into 1.0 mg/mL BSA or 1.0% human serum (human male AB plasma, Sigma, UK).

## Results and Discussion

### Peptide Sequence Selection and Binding Performance

**Figure 2:**
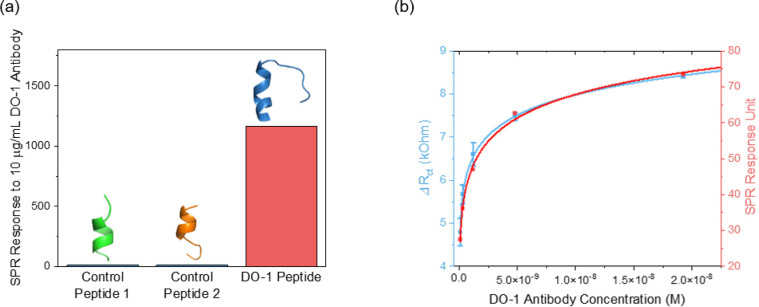
(a) SPR peptide specificity assessments of the synthesized DO-1 sequence (CQETFSDLWKLLPENNVL) and control peptides, when exposed to 10 μg/mL DO-1 antibody. Control peptide 1 (SPDDIEQWFT) was a nontarget p53 epitope (amino acids 46–55 on the p53 protein). Control peptide 2 (CPPPPEKEKEK) served as a non-p53 control. (b) Comparative binding affinity assessment between Con A and DO-1 antibody as assessed by electrochemical impedance spectroscopy (EIS, blue) and SPR (red). SPR analyses were at Con A-saturated gold chips, with a BSA-saturated surface as the reference. EIS measurements were conducted at Con A-modified SPCEs in a 5 mM [Fe(CN)_6_]^3/4–^ PBS solution (pH = 7.4). The impedimetric and SPR responses were both fitted to Langmuir–Freundlich isotherms (*R*^2^_SPR_ = 0.993 vs *R*^2^_EIS_ = 0.999, *K*_d,ConA-DO-1antibody_ = 13.6 ± 2.3 nM from the plot mean). Impedimetric error bars represent the standard deviation from three individual electrodes (*n* = 3).

As stated above, the DO-1 antibody targets a specific epitope found within amino acids 20–25 (SDLWKL) of the human p53 protein TAD domain.^12,29^ This sequence is part of the antigen α-helix (QETFSDLWKL) as elucidated crystallographically (PDB: 8F2I). To emulate the native antigen, we selected a peptide sequence (DO-1 peptide, CQETFSDLWKLLPENNVL) as a functional mimic. As illustrated in Figure S2, the binding affinity between this synthetic peptide and the DO-1 antibody was initially assessed using surface plasmon resonance (SPR) and observed to be comparable to the full recombinant protein (*K*_d,peptide_ = 0.34 nM *vs K*_d,protein_ = 0.12 nM).^29^ A validation of sequence specificity was carried out by assessing the SPR response of peptide-modified interfaces toward 10 μg/mL DO-1 antibodies (see experimental section, SI), with the synthesized sequence cross compared to two control peptides: SPDDIEQWFT (control peptide 1, mimicking a nonbinding region of the p53 protein)^30^ and CPPPPEKEKEK (control peptide 2, a non-p53 related, nonfouling peptide). As demonstrated in Figure 2a, DO-1 antibody recruitment, at comparable peptide surface densities (∼100 ng/cm^2^), was >2 orders of magnitude higher with the synthesized sequence.

To assess antibody recruitment at the lectin-modified electrodes, Con A/DO-1 antibody interactions were mapped by SPR (see experimental section, SI) with a resolved affinity consistent with literature (Figure S3).^31^ Con A-modified electrode interfaces were prepared via overnight physisorption after plasma treatment (Figures S4 and S5).^32^ Continuous impedimetric analysis of these interfaces was consistent with high levels of stability (Figure S6). Its binding affinity for the DO-1 antibody aligned with that resolved by SPR (Figure 2b). A good level of antibody selectivity was confirmed (Figure S7), with markedly (<10%) lower responses to 1.0 mg/mL levels of nonspecific protein, i.e., HSA and BSA.

### Dual-Functionalized IONPs

Low-polydispersity IONPs were prepared by hydrothermal synthesis (see Experimental Section) with a hydrodynamic size of 242.1 ± 2.1 nm (PDI < 0.06, Figures S8 and S9, SI).^33^ The Fourier-transform infrared spectroscopy (FTIR) and powder X-ray diffraction (PXRD) characteristics of these were as expected (Figures S10 and S11, SI).^33^ The antigen-mimic interfaces were constructed by immobilizing the DO-1 peptide, along with HRP, through standard active ester coupling (as noted above),^34^ a process tracked by dynamic light scattering (DLS) and zeta-potential (Figure S8) assessments. HRP and DO-1 peptide surface densities were quantified by 2,2′-azino-bis(3-ethylbenzothiazoline-6-sulfonic acid) (ABTS) UV–vis and micro-BCA assays, respectively (see experimental section, SI).^35^ The peptide coverage was demonstrably tunable, from 6 to >80% of the geometric surface area (Figure S12), with HRP levels diminishing at the higher end of this range (presumably through competition). This tunable composition was observed to have a direct effect on assay performance (see below). We expect any overestimation of peptide surface coverage, by the assumption of negligible displacement by HRP, to be minimal (Figure S12, SI).

### Amplified Electrochemical p53 Antibody Quantification

**Figure 3:**
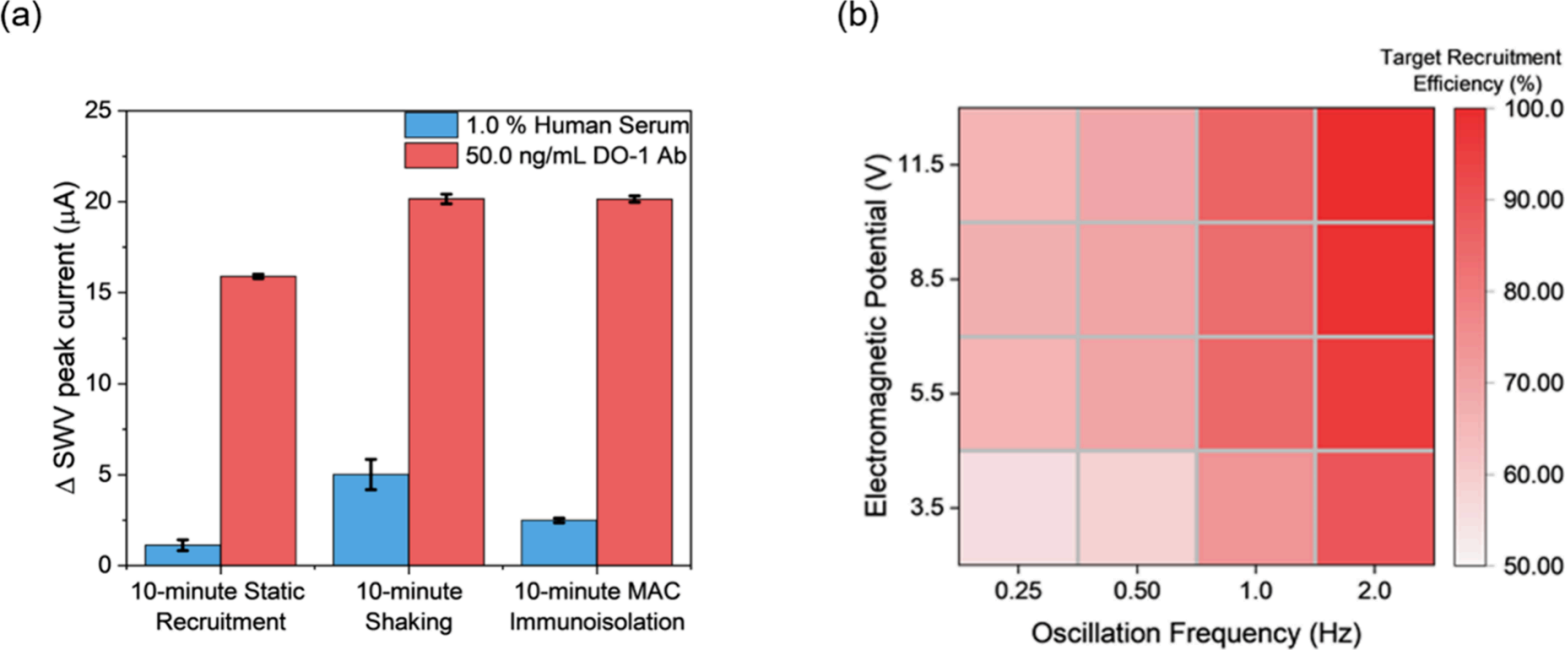
(a) Comparative SWV voltammetric responses to 50.0 ng/mL DO-1 antibody versus serum under three incubation conditions (10 min static recruitment), 10 min shaking, or magnetic mixing (10 min MAC immunoisolation) demonstrating the advantages associated with magnetic field-assisted recruitment. Static recruitment involved incubating samples with bioreceptive IONPs for 10 min. Shaking employed a standard lab vortex mixer. MAC immunoisolation was performed within a microfluidic channel (see Experimental Section) under an optimized AC magnetic field (see below). (b) The effect of nanoparticle oscillation frequency (up to 2.0 Hz) and AC magnetic field strength (adjusted by electromagnetic potential) on microfluidic DO-1 antibody immunoisolation. Recruitment efficiency was normalized to 100% using the SWV current response of bioreceptive IONPs capturing 1 ng/mL DO-1 antibody as acquired under optimized microfluidic isolation conditions (i.e., electromagnetic potential = 11.5 V; nanoparticle oscillation frequency = 2.0 Hz; flow rate = 50 μL/min).

A 100 μL aliquot of 10 mg/mL peptide-modified IONPs was utilized for DO-1 antibody recruitment, initially from buffer. Prior studies have demonstrated that target recruitment at planar receptor surfaces is commonly limited by sluggish binding kinetics and undesired fouling in serum samples,^15^ necessitating either prolonged incubation and/or antifouling strategies. Herein, significant fouling (>25% of specific response) in serum-spiked samples under static recruitment was noted, following a 1 h incubation (Figure 3a). To enhance recruitment efficiency and specificity, we employed a microfluidic configuration paired with a programmable AC electromagnetic field (Figure S1; see Experimental Section for details).^25^ A time-course study (Figure 3a and Figure S14) demonstrated that MAC immunoisolation accelerates both target recruitment and specificity (background response reduced by 60%, shown in Figure S15). A comparative 10 min vortex shaking, which also promotes mixing, similarly accelerates target recruitment (Figure 3a) but does so with much higher (∼100% more) levels of nonspecific binding. These results support the value of MAC-supported target recruitment. It was apparent that, as noted in a previous work,^25^ capture efficiency was a sensitive function of particle oscillation frequency, field strength, and microfluidic flow rate; and was thereafter optimized at 2 Hz, 11.5 V, and 50 μL/min. Increasing the particle oscillation frequency significantly enhanced the resultant target recruitment efficiency (Figure 3b) but was herein capped at 2.0 Hz by both equipment limits and concerns about magnetic nanoparticle hyperthermia at higher frequencies and any downstream associated protein denaturing.^36^

**Figure 4:**
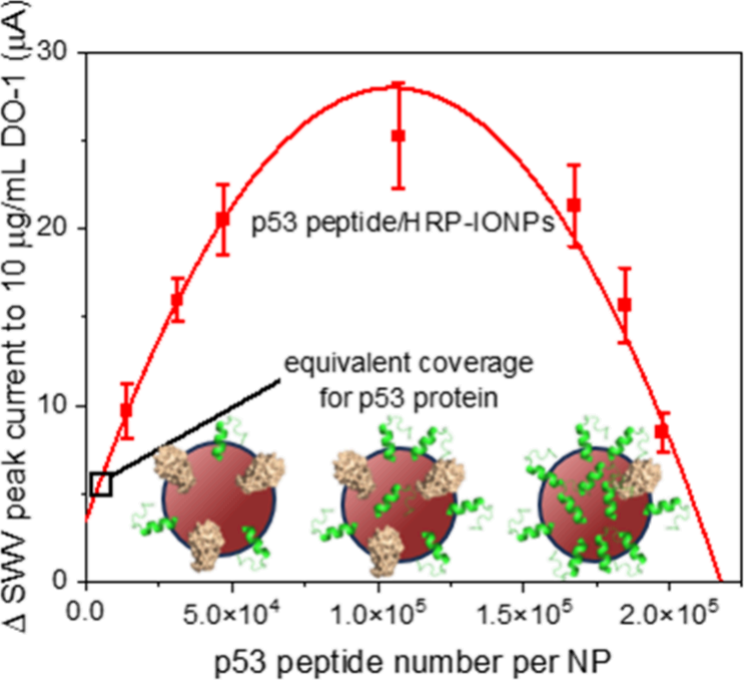
SWV assessment of the effect of IONP surface composition on sensing performance when exposed to 10 μg/mL DO-1 antibody. The red line represents a quadratic correlation between IONP peptide coverage and the resulting electrochemical signal with an *R*^2^ of 0.95. The black square indicates the equivalent surface coverage and peak current of the full antigen protein. SWV measurements were conducted in 100 μL of TMB substrate solution, ranging from +1.0 V to −0.5 V (vs Ag/AgCl) with an amplitude of 20 mV, a potential step of 5 mV, and a frequency of 50 Hz. All error bars represent the standard deviation across three individual recruitments (*n* = 3).

Following immunoisolation, the antibody–IONP complexes were collected by pipet and manually transferred to the Con A-modified SPCEs (Figure 1; see Experimental Section for detail). In the presence of TMB and H_2_O_2_, the SWV-generated current reported sensitively on the concentration of DO-1 (Figure S16)^37^ As noted, the nanoparticle receptor (peptide) and signal generating (HRP) surface composition modulated the sensing performance (signal generated per concentration of target), necessitating optimization. This was, specifically, a quadratic trend (see Figure 4 and eqs S1–S9, SI) reflective of the balance between target capture probability and HRP-based signal generation (decreasing the HRP load at very high peptide surface densities) (Figure S12). Optimal antibody responses were obtained at ∼50% geometric peptide coverage (1.0 × 10^5^ peptides per IONP), prepared using a mixture of 5.00 μg/mL p53 peptide and 50.0 μg/mL HRP.

**Figure 5:**
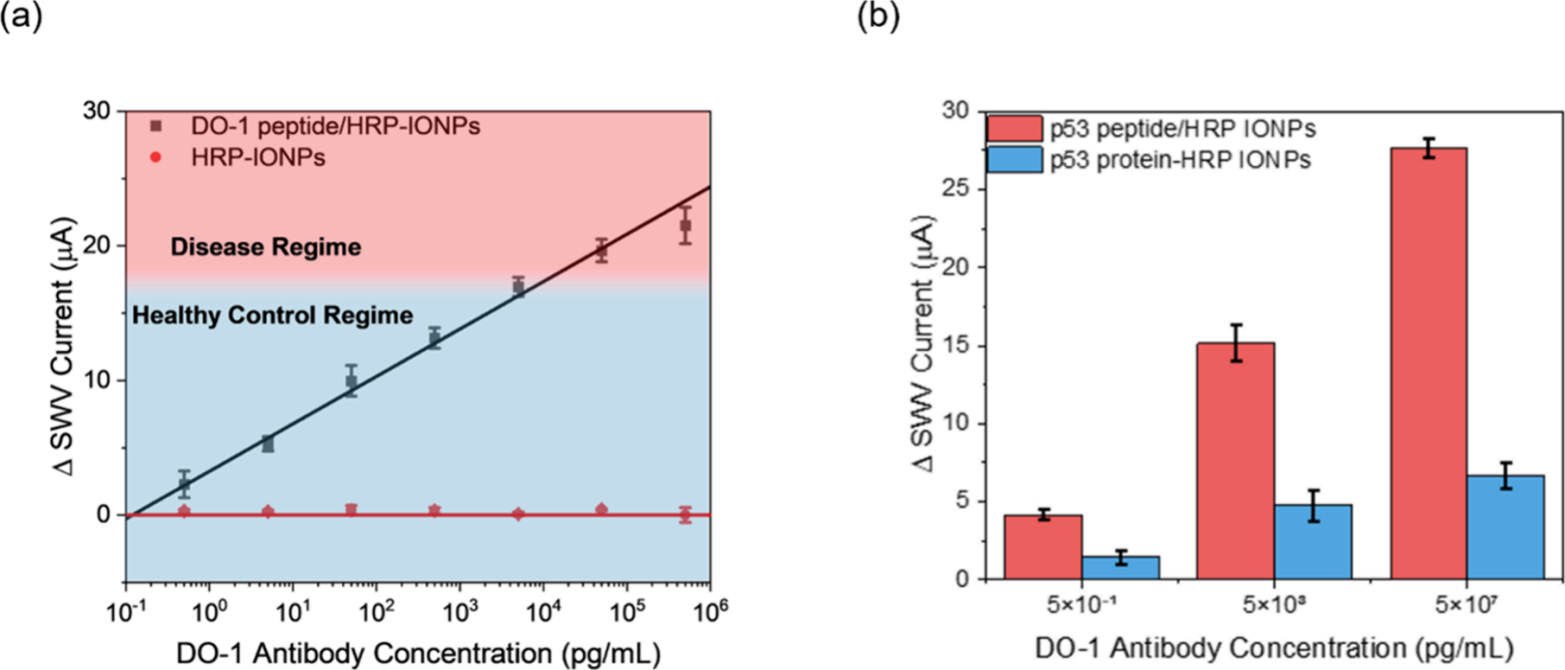
(a) Voltammetric response of Con A-modified SPCE interfaces to the target DO-1 antibody as recruited by DO-1 peptide/HRP-IONPs (black) and control, nonreceptive HRP-IONPs (red), respectively (black line representing a linear fitting, *R*^2^ = 0.99). Two regimes (healthy control and disease) were defined using literature values,^38^ extrapolated to the calibration curve. (b) Comparison of sensor response to target antibody (immunoisolation under the specified optimized MAC conditions) for the target specific peptide (i.e., DO-1 peptide) and full antigen capture particles. All error bars depict the standard deviation from three individual electrodes (*n* = 3).

Following immunoisolation using the optimized particle surface composition, the captured DO-1 antibody concentration was electrochemically assessed by SWV at Con A arrays. As shown in Figure 5a, a dynamic range spanning from 5.0 pg/mL to 0.5 μg/mL was noted, with good repeatability (standard deviation <5% across the full dynamic range). The limit of detection (LoD) was 1.0 pg/mL (8.3 fM), comfortably surpassing clinical needs. Significantly, the assay supported by the peptide-based nanoparticles was observed to be 400% more sensitive than that observed with the full-length p53 protein (Figure 5b) under equivalent preparation and analytical conditions (i.e., bioreceptor and nanoparticle concentrations and incubation time). We attribute this to the higher bioreceptor surface density (50 vs 5% geometric surface area; as confirmed by the micro-BCA assay) and the correspondingly increased probability of target capture (Figure 4).^18,19^

**Figure 6:**
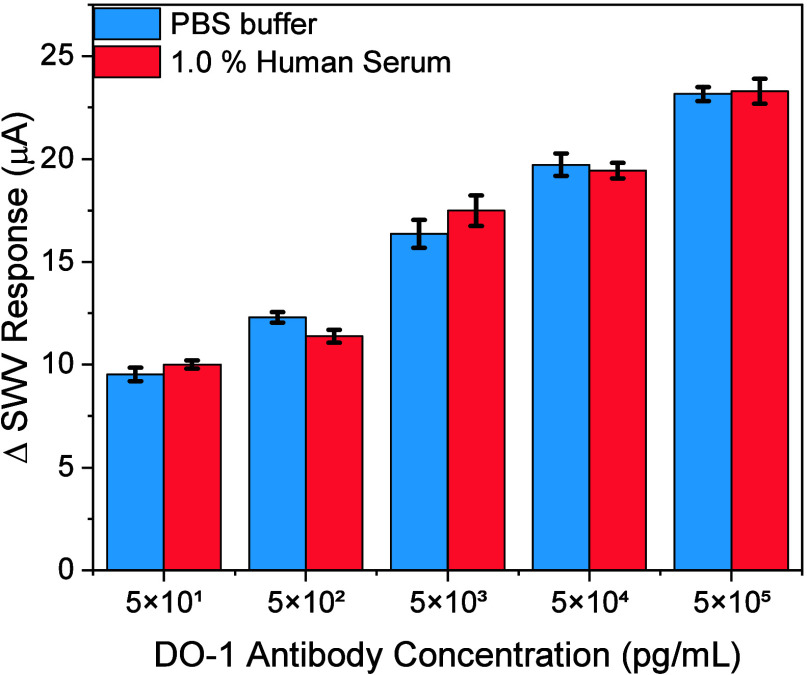
Recovery analysis across clinically relevant concentrations of DO-1 antibody spiked into 100 μL of 1.0% human serum. All error bars represent the standard deviation across three individual electrodes (*n* = 3).

Assay specificity was assessed against 1.0 mg/mL BSA, HSA, 1.0% human serum, and 10.0 μg/mL immunoglobulin G (antibody control), with negligible voltammetric responses (<10% of that presented by 50 ng/mL DO-1 antibody) obtained under equivalent MAC immunoisolation conditions (Figure S17). To evaluate the performance in spiked samples, recovery tests were performed in 1.0 mg/mL BSA and diluted human serum. These tests yielded an average recovery of 96.7 ± 3.2% in BSA (Figure S18,*n* = 3) and 100.7 ± 5.0% in human serum (Figure 6, *n* = 5), indicative of robustly selective target recruitment.

## Conclusions

Anti-p53 autoantibodies, triggered by the abnormal accumulation of p53 proteins, can provide insight into cancer progression and prognosis, serving as important biomarkers for early diagnosis. To improve the efficacy with which, these valuable targets can be recruited from solution and presented to scalable and readily prepared sensors, we have herein employed a peptide recognition element on magnetically manipulatable nanoparticle–antigen mimics. These peptide-functionalized particles exhibited a high sequence specificity and antibody binding affinity. When integrated within 3D-printed microfluidic configurations, they can be field-programmed to isolate the target within a few minutes, with a lower background than that under conditions of static target recruitment from complex samples. We noted a quadratic distribution between target-generated voltammetric response and DO-1 peptide nanoparticle surface density, and assign this to a balance between capture probability and signal generation. It is significant that the peptide-modified nanoparticles supported an electrochemical response that is 400% more sensitive per unit target concentration than that of the equivalent full antigen particles. The lectin-modified carbon arrays promoted the delivery of captured targets to sensors and did this with good levels of specificity, ultimately underpinning assays spanning a six-order dynamic range and subfemtomolar sensitivity that translates well to serum-based analyses.

## Supporting Information Available

Experimental details, materials, instruments, relevant protocols, nanoparticle characterisation, SPR immunoanalyses, electrochemical methods, and the derivation of relevant equations (PDF)
