# Supplementary material for: Antigen-Mimic Nanoparticles in Ultrasensitive on-Chip Integrated Anti-p53 Antibody Quantification
Source: ACS Sens. 2024 Mar 5;9(3):1475–81. doi: 10.1021/acssensors.3c02568 (PMC10964233; doi:10.1021/acssensors.3c02568)
Supplement: Supplementary file 1 — se3c02568_si_001.pdf [file se3c02568_si_001.pdf]

SUPPORTING INFORMATION

Antigen-mimic Nanoparticles in Ultrasensitive On-Chip Integrated Anti-p53 Antibody Quantification

Shaoyu Kang, Daohe Yuan, Robert Barber, Jason J. Davis*

* Corresponding author

Email: Jason.davis@ chem.ox.ac.uk

Department of Chemistry, University of Oxford, South Parks Road, Oxford, OX1 3QZ, U.K.

Table of Contents

Experimental Details S-2
Figure S1. The schematic depiction of the microfluidic immunoisolation configuration S-4
Figure S2. SPR response assessment of DO-1 antibody to p53 peptide and p53 full protein S-4
Figure S3. SPR response assessment of DO-1 antibody to Concanavalin A binding affinity S-5
Figure S4. Cyclic voltammetry comparison between GC disc electrode, SPCE and plasma cleaned SPCE array S-5
Figure S5. Impedimetric response of DO-1 antibody to Concanavalin A binding affinity S-6
Figure S6. EIS baseline stability of Concanavalin A-modified SPCE S-6
Figure S7. EIS & SPR specificity assessment of Concanavalin A S-7
Figure S8. DLS and zeta-potential measurement of bio-receptive IONPs S-7
Figure S9. TEM images of IONPs S-8
Figure S10. FTIR spectrums of IONPs and HRP-p53 IONPs S-8
Figure S11. PXRD spectrums of IONPs S-9
Figure S12. p53 peptide and HRP surface coverage on IONPs. S-9
Figure S13. Flow rate impact on microfluidics capture efficiency S-10
Figure S14. Time-course comparative study of DO-1 Ab recruitment in MAC immunoisolation and static incubation S-10
Figure S15. Time-course comparative study of human serum fouling in MAC immunoisolation and static incubation S-11
Figure S16. SWV response of bio-receptive IONPs towards varying concentration of DO-1 ..... S-11
Figure S17. SWV response specificity assessment of bio-receptive IONPs S-12
Figure S18. Recovery assessment of DO-1 antibody spiked in BSA and Human Serum matrices S-12
Equation S1-S9. Bioreceptor/redox probe surface coverage impact on electrochemical response S-13
Figure S19. Bioreceptor surface coverage impact on current response S-14
Reference S-14

EXPERIMENTAL DETAILS

Materials and Instruments: Sodium chloride, disodium hydrogen phosphate, potassium chloride, potassium dihydrogen phosphate, sodium acetate, trisodium citrate, ethylene glycol, anhydrous iron (III) chloride, 2,2′-Azino-bis(3-ethylbenzothiazoline-6-sulfonic acid) diammonium salt (ABTS, ≥ 98%), hydrogen peroxide solution (30% w/w in water), peroxidase from horseradish (HRP), albumin from human serum (HSA), IgG (from bovine serum, ≥ 95%), and bovine serum albumin (BSA) were purchased from Sigma (UK). Tween-20, and the Pierce™ Micro BCA protein assay kit were purchased from ThermoFisher Scientific (UK). TMB high sensitivity substrate solution was purchased from Biolegend, Inc (UK). Concanavalin A was purchased from Cayman Chemistry Company. Monoclonal p53 antibody (DO-1) was purchased from Santa Cruz Biotechnology (UK). p53 protein was purchased from Proteintech Group (UK). p53 peptides (DO-1 peptides, H-CQETFSDLWKLLPENNVLSPL-OH; AB-2 peptide, H-SPDDIEQWFT-OH) and control peptide (CPPPPEKEKEK) were sourced from ISCA Biochemicals Ltd. All chemicals were used without further purification.

Fourier Transform Infrared Spectra (FT-IR) were recorded using an FT-IR spectrophotometer (IRTracer-100, Shimadzu, Japan). Surface Plasmon Resonance (SPR) was conducted on a Biacore SPR instrument (Biacore X100, Cytiva). The nanoparticle hydrodynamic size and Zeta potential were determined using a Zetasizer (Malvern Panalytical, UK). Microwell plate BCA assays were analysed using a plate reader (iMark Microplate Absorbance Reader, Bio-Rad, Laboratories, Inc., UK). The microfluidic configuration consisted of custom 3D-printed cells, an injector, a syringe pump, and a pair of electromagnets with programmable power supply. TEM images were obtained from a Transmission Electron Microscope (FEI Tecnai T12, 120 kV, USA). Plasma cleaning was conducted in a low-pressure plasma system (FEMTO, Diener Electronic, Germany).

Electrochemical measurements were carried out in a 10 mM PBS buffer solution with a pH of 7.4, unless otherwise specified. Electrochemical impedance spectroscopy (EIS) was executed from 100 kHz to 0.1 Hz, encompassing 40 frequencies, with a sinusoidal AC perturbation of 5 mV in a 5 mM ferrocyanide PBS solution. During this process, the DC potential was held constant at the half-wave potential of ferrocyanide (+ 0.21 V, relative to Ag/AgCl). The charge transfer resistance (R_{ct}) was obtained from the diameter of the inflection point in the Nyquist plot. Square-wave voltammetry (SWV) was performed from + 1.0 V to – 0.5 V (relative to Ag/AgCl) with an amplitude of 20 mV, potential step of 5 mV, and a frequency of 50 Hz.

Micro-BCA assay for p53 peptide surface coverage determination: A series of 150 μL standards of p53 peptide, with concentrations ranging from 0.5 mg/mL to 40.0 mg/mL were prepared in 10 mM PBS buffer at a pH of 7.4. Two 1 mg/mL IONP samples were prepared, namely a p53 peptide-IONPs (conjugated using the optimised peptide coverage) and bare IONPs controls. Next, 150 μL of each standard and sample were dispensed into individual wells of a microplate. An equal volume of the Micro-BCA working solution was added to each well, followed by thorough mixing on a plate shaker for 30 seconds. The microplate was then transferred to an incubator set at 37 °C for a two-hour incubation. After cooling the plate to room temperature, a magnet was used to concentrate the IONPs to one side of the wall (observed to reduce the scattering error in the subsequent measurements). Subsequently, the absorbance of the solutions was measured at 590 nm using a plate reader. With calibration established, the difference in absorbance between the p53 peptide-IONPs and the bare IONPs was utilised to estimate the surface coverage of the p53 peptide on the IONPs. The error bars were estimated based on three individual measurements (n = 3). Notably, the surface coverage of p53 peptides, due to the co-immobilisation of HRP, is supposed to be overestimated, particularly at high levels of p53 peptide coverage.

ABTS assay for HRP surface coverage quantification: A 1.0 mM solution of ABTS (2,2'-Azino-bis(3-Ethylbenzthiazoline-6-Sulfonic Acid)) was prepared in a PBS buffer (pH = 5.0). This solution was then mixed with 5.0 μL of a serially diluted HRP solution, and the UV-Vis absorption monitored at 415 nm. Once a stable baseline was achieved, a 0.3% (w/w) hydrogen peroxide solution (10.0 μL) was introduced and the solution left undisturbed for in two minutes before the absorption at 415 nm was recorded to construct a calibration curve. Following that, the absorption at 415 nm was measured two minutes after introducing 5.0 μL of HRP/p53-IONPs into the ABTS-hydrogen peroxide mixture. The difference in absorption at 415 nm between the HRP/p53-IONPs and bare IONPs was used to estimate

the surface coverage of HRP. The error bars were estimated from the standard deviation of three individual measurements (n = 3). The enzymatic activity of these nanoparticle-bound HRPs is expected and assumed to be similar to that of the free form in solution.[1]

SPR assessments of biomolecular binding affinity and peptide sequence specificity: SPR gold chips (SIA kit Au, Cytiva) were first rinsed with ethanol, then deionised water several times before submerging the chips in piranha acid (composition as above) for 15 minutes. The chips were then rinsed thoroughly with deionized water. SPR assessments were conducted at room temperature (298 K) at a flow rate of 30 μL/min. The running buffer was a 10 mM PBS solution (pH = 7.4). In assessing the antibody binding affinity of the p53 peptide (*i.e.*, DO-1 peptide) and p53 protein, peptide-saturated or protein-saturated interfaces were prepared by a 1-hour injection with 100 μg/mL samples, respectively. The SPR sample channel coverage was estimated using the standard Biacore working protocol where surface concentration = 1.0·ΔRU pg/mm^2, with ΔRU being the difference in response units between the sample-injected and control channels, the latter exposed to injections of the running buffer. A full SPR sensorgram was constructed by injecting a series of increasing concentrations of DO-1 antibody solution (from 39.1 ng·mL^{-1} to 10.0 μg·mL^{-1}, 4-fold dilution). The antibody binding affinity (K_d) for the p53 peptide or p53 protein was determined respectively from a Langmuir-Freundlich isotherm fitting. Similarly, the SPR affinity assessment between the DO-1 antibody and Con A was conducted on a Con A-saturated SPR gold chip. To assess peptide sequence specificity, a 100 μg/mL peptide solution (DO-1 peptide, control peptide 1 or control peptide 2) was immobilised onto the SPR gold chip following standing protocols (active ester coupling on a 3-Mercaptopropionic acid-saturated surface or direct cysteine tethering). Following a blocking procedure (30-min incubation with 1% BSA), the peptide sequence specificity was assessed by exposing the modified channel to a 10 μg/mL DO-1 antibody solution.

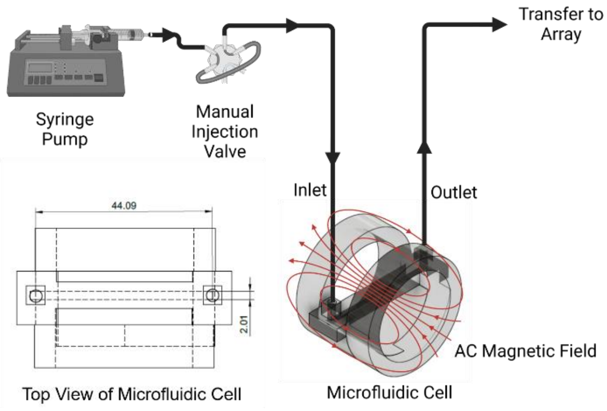

Figure S1. Schematic depiction of the microfluidic immunoisolation configuration, comprising a microfluidic channel, syringe pump, manual injection valve, and a pair of programmable electromagnets. The microfluidic channel was prepared by 3D printing (Elegoo Mars Pro 405 nm curing transparent resin; dimensions in millmeters of the channel given in the top view drawing), with two rings on both sides to host the electromagnets. The sample and running buffer were delivered at a controlled flow rate by an automated syringe pump. Flow rates of up to 200 $\mu L \cdot min^{-1}$ were accessible. The AC magnetic field generated by the pair of electromagnets was tuned by adjusting the oscillation frequency and electromagnetic field strength (adjusted by potential, up to 12 V). Following immunoisolation, the resulting antibody-IONP complexes were manually transferred onto an electrode array by pipette for subsequent electrochemical assay.

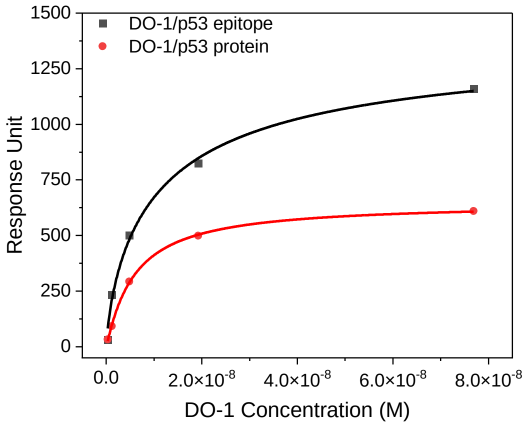

Figure S2. SPR response assessment of varying concentrations of DO-1 antibody on p53 peptide (black) and p53 full protein (red) saturated gold chips. SPR was conducted at room temperature (298 K) with a flow rate of 30 mL/min. A 10 mM PBS solution (pH = 7.4) served as the running buffer.

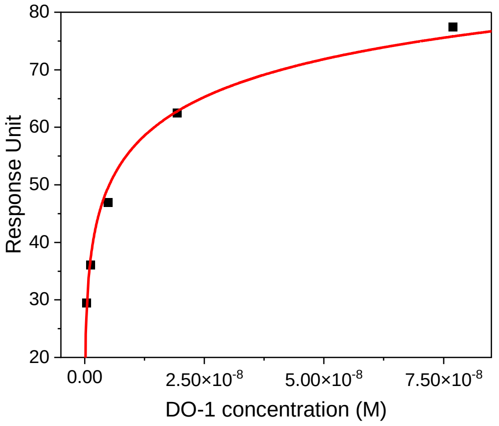

Figure S3. SPR assessment of the DO-1 antibody recruitment at Concanavalin A-saturated gold chips, using a BSA-saturated surface as reference. SPR was conducted at room temperature (298 K) with a flow rate of 30 µL/min. The K_d of the Langmuir- Freundlich isotherm was determined to be 15 nM with an R^2 of 0.984.

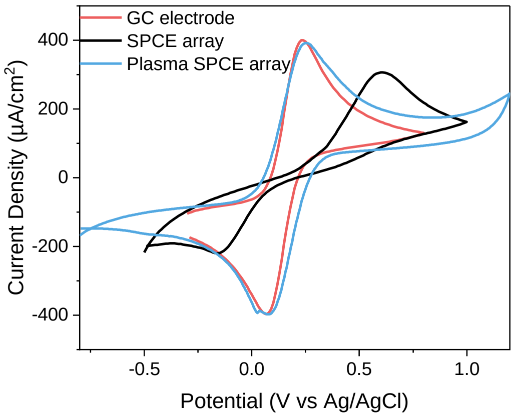

Figure S4. Cyclic voltammetry comparison between a GC disc electrode, a SPCE and a plasma cleaned SPCE array. CV measurements were conducted in a 5 mM $[Fe(CN)_6]^{3/4-}$ PBS solution (pH = 7.4) at a scan rate of 25 mV/s. In assessing peak separation, peak current ratios in CV, and the charge transfer resistance in EIS, it is evident that the electrochemical performance of plasma-treated SPCE electrodes is comparable to that of GC disk electrodes, supporting the applicability of the low-cost plasma cleaned SPCE array in electroanalysis.

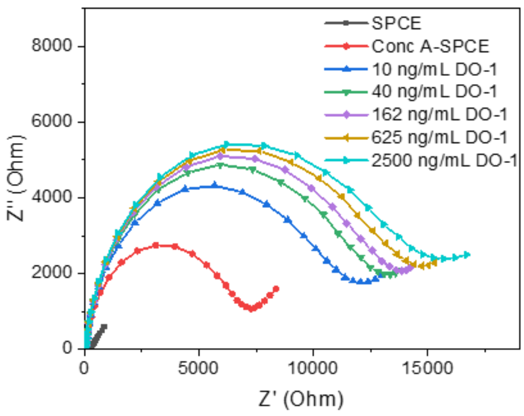

Figure S5. Impedimetric Nyquist plots of a plasma cleaned and Con-A modified screen-printed carbon electrode (SPCE, black), before (red) and after exposed to various concentrations of DO-1 antibody, measured in a 5 mM $[Fe(CN)_6]^{3/4-}$ PBS solution (pH = 7.4).

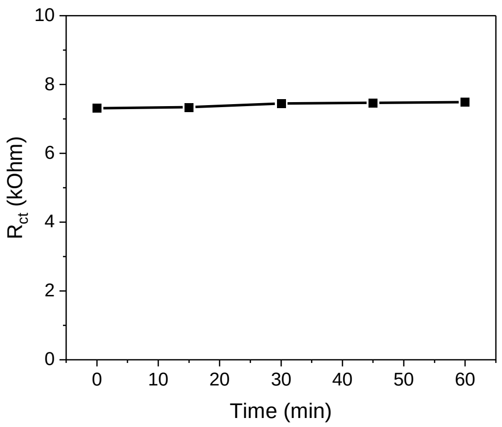

Figure S6. EIS baseline stability of the Con A-modified SPCE electrode in a 5 mM $[Fe(CN)_6]^{3/4-}$ PBS solution (pH = 7.4). Over one-hour of repeated measurements, a shift of less than 3% was observed, i.e a high levels of baseline stability is apparent.

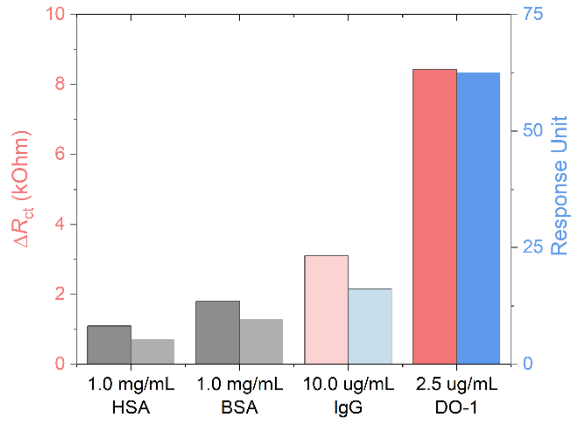

Figure S7. SPR (blue) and impedimetric (red) assessments of Con A-modified surface specificity to a 2.5 µg/mL DO-1 antibody (target), 10.0 µg/mL IgG (antibody control) and nonspecific proteins (*i.e.*, 1.0 mg/mL HSA and BSA). SPR was conducted at room temperature (298 K) on a p53 peptide-saturated gold chip with a flow rate of 30 µL/min. The running buffer was a 10 mM PBS solution with a pH of 7.4. EIS measurements were performed on a Con A-modified SPCE electrode interface, measured in a 5 mM $[Fe(CN)_6]^{3/4-}$ PBS solution (pH = 7.4). Both sets of results indicated that Con A specifically recruits antibodies (DO-1 and IgG), particularly to DO-1 antibodies, while the responses to BSA and HSA were negligible (< 10%).

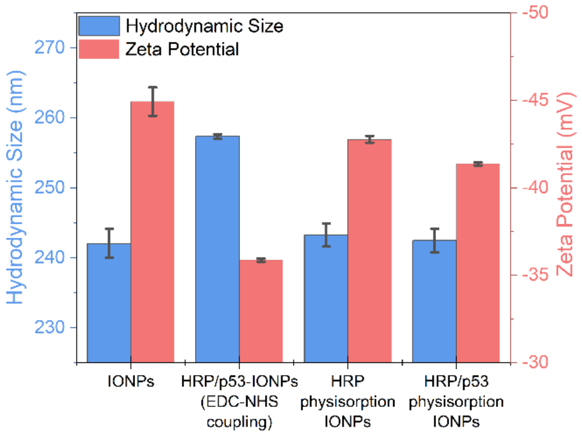

Figure S8. DLS (blue) and Zeta potential (red) measurements of IONPs, and HRP-p53-IONPs prepared by EDC-NHS coupling (with simple physisorption shown as a comparison). These measurements were conducted at room temperature, with IONPs samples were dispersed in a 10 mM PB buffer at a pH of 7.4. All error bars represent the standard deviations across three individual measurements (n = 3). The average hydrodynamic size of IONPs was approximately 242.1 ± 2.1 nm. After conjugation, an increase in hydrodynamic size of around 15 nm was noted, along with a decline in Zeta potential (~ 10 mV). Both changes attributed to the immobilisation the HRP onto the IONPs (8 nm in size; an isoelectric point of 9).[2] The minimal changes (< 1 nm in size; 3 mV in Zeta potential) observed in the physisorption control samples may be ascribed to low modification efficiency by this method.

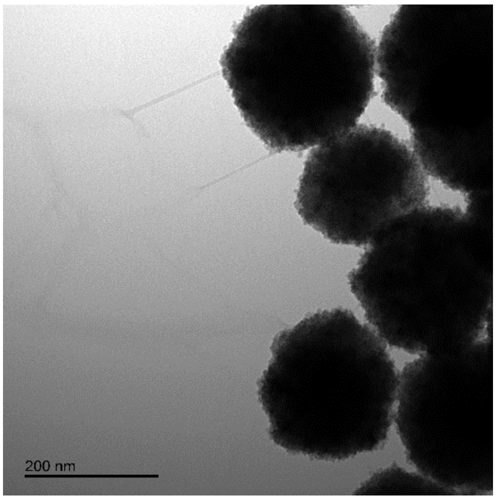

Figure S9. TEM image of citrate-chelated IONPs, showing an average size of 200 nm.

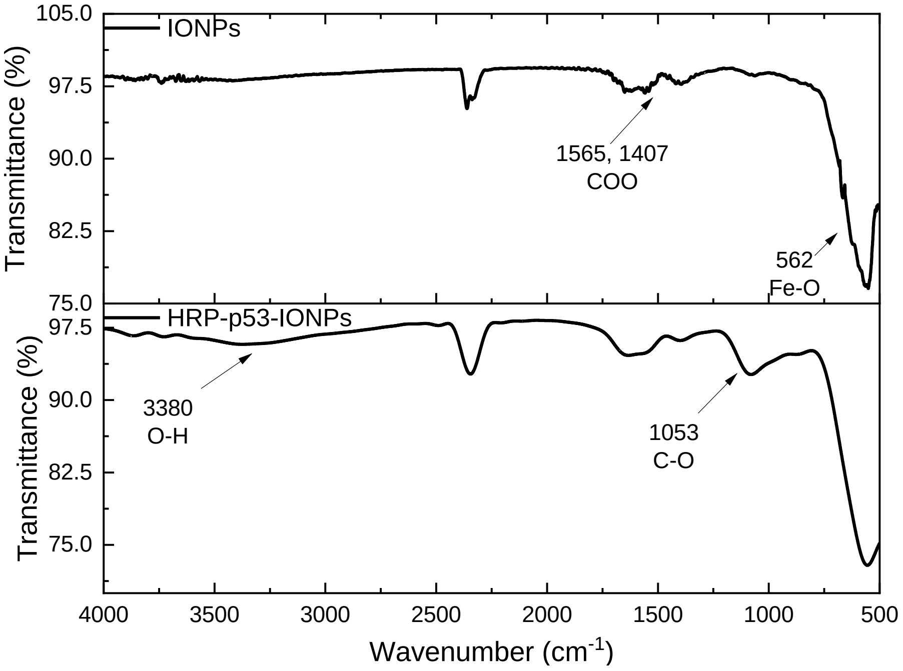

Figure S10. FT-IR spectrum of the native and HRP-p53 peptide-IONPs. Three characteristic IR peaks at 1565 cm^{-1}, 1407 cm^{-1} and 562 cm^{-1} were noted, corresponding to the C=O asymmetric/symmetric stretching and Fe-O stretching, respectively. Two additional characteristic peaks at 3380 cm^{-1} and 1053 cm^{-1} in the HRP-p53 peptide-IONPs samples were attributed to O-H and C-O stretching, respectively, observations all consistent with the peptide/HRP mixed particle interface. The peak around 2349 cm^{-1} is assigned to the asymmetric stretching of CO_2 from the atmosphere.

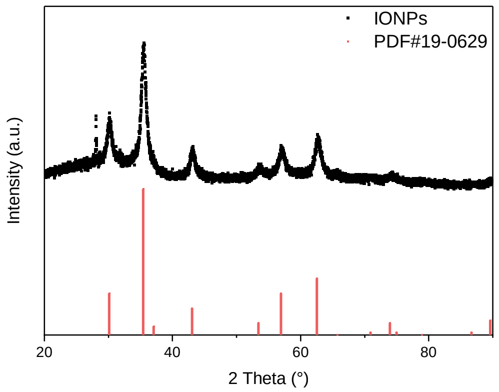

Figure S11. Powder X-ray diffraction (PXRD) pattern of hydrothermal synthesised iron oxide nanoparticles (upper, Cu $K\alpha_1$). The lower ticks correspond to standard powder diffraction file for Fe_3O_4 (PDF#19-0629).

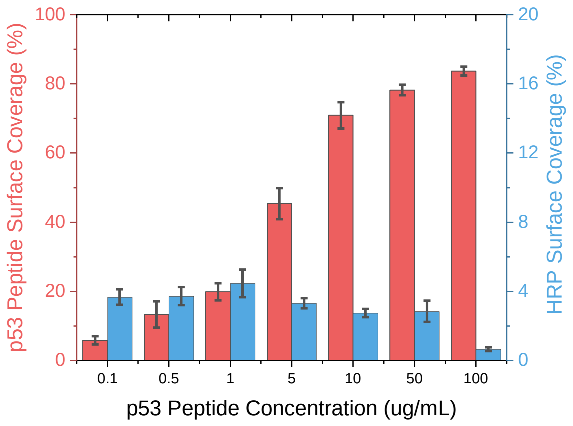

Figure S12. Surface coverage of p53 peptide (red) and HRP (blue) on IONPs. The ratio of p53 peptide to HRP surface coverage was adjusted by varying the p53 peptide concentration in the preparation solution from 0.1 μg/mL to 100 μg/mL. p53 peptide surface coverage was determined from a micro-BCA assay, while the HRP surface coverage was quantified via the ABTS UV-Vis. All error bars represent the standard deviations across three individual measurements (n = 3). As the p53 peptide surface coverage increased, that of the HRP generally decreased. Specifically, the coverage for the DO-1 peptide was tuneable, ranging from 5.9% to 83.7%, whereas the HRP coverage was less than 5% of the IONPs' geometric surface area, and diminished further at higher peptide surface coverages.

Althrough the co-immobilisation of HRP and p53 peptide may result in a possible overestimation of peptide surface coverage, especially in the presence of competitive HRP conjugation, the >order of magnitude difference in peptide (> 50%) HRP (< 5%) coverages will make the quantitative influence of this small. The quantification of nanoparticle-bound HRPs with the ABTS UV-Vis assay assumes enzymatic activity to be the same as that in solution; this is areasonable assumption.[13]

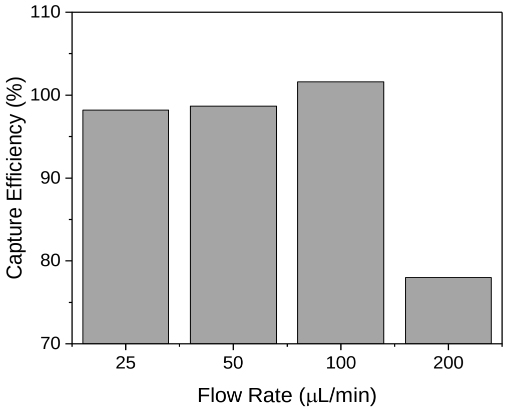

Figure S13. The influence of flow rate on the DO-1 antibody capture efficiency using an electromagnetic potential of 11.5 V and nanoparticle oscillation frequency of 2 Hz. Recruitment efficiency was normalised to 100% using the SWV current response of bio-receptive IONPs capturing 1 ng/mL DO-1 antibody under optimised microfluidic conditions (*i.e.*, electromagnetic potential = 11.5 V; nanoparticle oscillation frequency = 2.0 Hz; flow rate = 50 µL/min). Capture efficiency improved marginally but significantly as the flow rate increased from 25 µL/min to 100 µL/min, a phenomenon attributed to increased analyte flux. At 200 µL/min, capture falls, most likely due to a reduced contact time between the IONPs and flowing target samples. To avoid excessive pressure and aid the retention of immuomodified IONPs within the microfluidic channel, the optimal flow rate for DO-1 antibody recruitment was determined to be 50 µL/min.

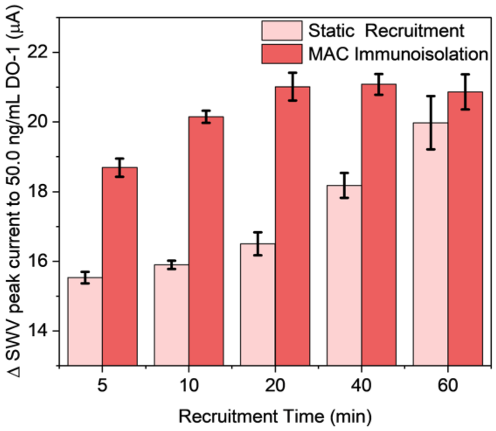

Figure S14. Comparative SWV voltammetric responses to 50.0 ng/mL DO-1 antibody under static incubation and MAC immunoisolation, over various time intervals ranging from 5 minutes to 1 hour. MAC recruitement was conducted under optimised microfluidic isolation conditions (*i.e.*, electromagnetic current potential = 11.5 V; nanoparticle oscillation frequency = 2.0 Hz; flow rate = 50 µL/min). Results demonstrate that MAC recruitement accelerates target recuirtment, reaching a maximun after10-minutes, compared with a 1-hour static incubation.

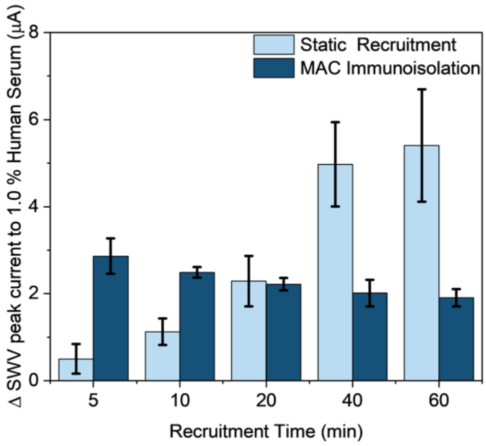

Figure S15. Comparative SWV voltammetric responses to 1.0 % human serum under static incubation and MAC immunoisolation, over various time intervals ranging from 5 minutes to 1 hour. MAC recruitement was conducted under optimised microfluidic isolation conditions (*i.e.*, electromagnetic current potential = 11.5 V; nanoparticle oscillation frequency = 2.0 Hz; flow rate = 50 μL/min). The results clearly show that non-specific fouling increases with prolonged static incubation, while it remains low under MAC immunoisolation under continuous flow.

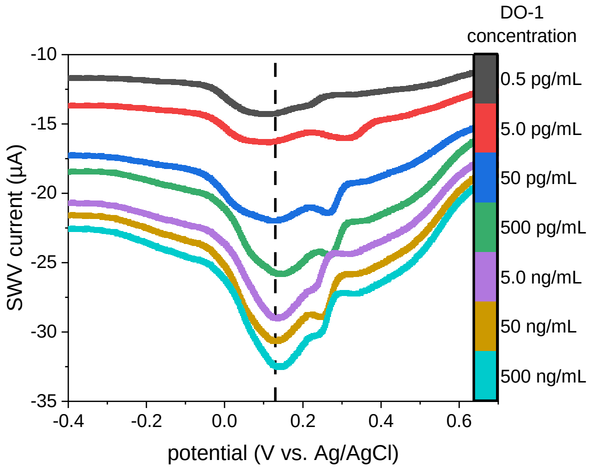

Figure S16. SWV response of bio-receptive IONPs towards varying concentrations of DO-1 antibody at Con A-modified SPCEs. SWV measurements were conducted in a 100 μLTMB substrate solution, scanning from +1.0 V to – 0.5 V (vs. Ag/AgCl) with an amplitude of 20 mV, a potential step of 5 mV and a frequency of 50 Hz after pre-isolation.

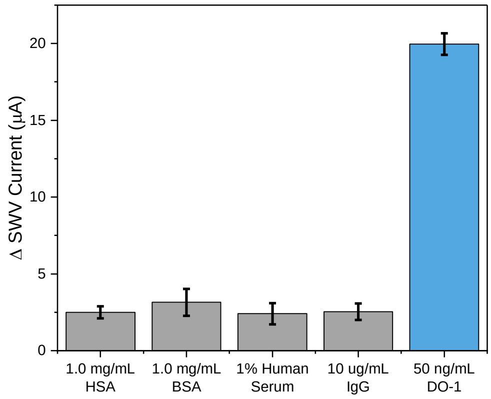

Figure S17. Specificity assessment of p53 peptide/HRP-IONPs towards a 5.0 ng/mL DO-1 antibody (target), 10.0 μg/mL IgG (antibody control), and nonspecific proteins (*i.e.,* 1.0 mg/mL HSA, BSA, and 1% human serum) following microfluidic pre-isolation, evaluated by the SWV current response. All error bars represent the standard deviations across three individual measurement (n = 3) Results indicated that p53 peptide modified IONPs specifically recruit DO-1 antibodies, with non target recruitment generating less than 10% of the signal.

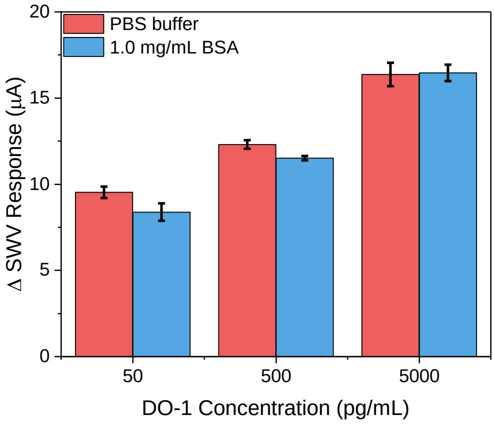

Figure S18. Recovery assessment of DO-1 antibodies spiked in 1.0 mg/mL BSA matrices. All error bars represent standard deviations across three individual electrodes (n = 3). The overall recovery for the assay recruiting targets from BSA-rich matrices was determined to be 96.7 ± 3.2%, indicating the robustness of the assay.

Bioreceptor/Redox Probe Surface Coverage Impact on Electrochemical Response

Since the nanoparticle receptor (peptide) and signal generating (HRP) surface composition was shown to modulate the sensing performance, this section discusses the influence of each on the voltammetric response. For the nanoparticle based amplified electrochemical assay, we assume the mass of iron oxide nanoparticles added on the electrode surface is: m_{NP}; Density of iron oxide nanoparticles is: ρ_{NP}; Size of iron oxide nanoparticles is: r_{NP}

Bioreceptor (p53 peptide) and electrocatalyst (HRP) were immobilised onto the nanoparticle surface by EDC-NHS coupling chemistry. We assume the surface coverage of bioreceptor (p53 peptide) on nanoparticle surface is: θ_a; while the surface coverage of electrocatalyst (HRP) on nanoparticle surface is: θ_b. The concentration of target (DO-1) in sample is noted as: c_{target}. The binding constant of bioreceptor-target (p53 peptide/DO-1) is: K_d

The number of iron oxide nanoparticles (n_{NP}) is:

$$n_{NP} = \frac{m_{NP}}{\frac{4}{3}\pi r_{NP}^3 \rho_{NP}} \tag{S1}$$

Surface area of iron oxide nanoparticles (S_{NP}) is given by:

$$S_{NP} = 4\pi r_{NP}^2 \cdot n_{NP} = \frac{3m_{NP}}{r_{NP}\rho_{NP}} \tag{S2}$$

The number of bioreceptor (p53 peptide) on NPs (n_a) is:

$$n_a = S_{NP} \cdot \theta_a \tag{S3}$$

After immunoislation the target (DO-1 antibody), an equilibrium of bioreceptor-target (p53 peptide/DO-1 antibody) complex is established as follows:

Bioreceptor-NPs + Target $\rightleftharpoons$ Target-Bioreceptor-NPs

The surface coverage of bioreceptor-target (p53 peptide/DO-1 antibody) complex ($\theta_{complex}$) on nanoparticles follows the Langmuir isotherm:

$$\theta_{complex} = \frac{n_{complex}}{n_a} = \frac{K_d \cdot c_{target}}{1 + K_d \cdot c_{target}} \tag{S4}$$

Assuming 1-to-1 binding of p53 peptide and DO-1 antibody, under no steric hindrance conditions, the ideal number of bioreceptor-target (p53 peptide/DO-1) complex ($n_{complex}$) is:

$$n_{complex} = \theta_{complex} \cdot n_a = \frac{K_d \cdot c_{target}}{1 + K_d \cdot c_{target}} \cdot S_{NP} \cdot \theta_a \tag{S5}$$

The number of HRP (n_{HRP}) on the nanoparticles which generate the sandwich on the electrode can be calculated as:

$$n_{HRP} = n_{complex} \cdot \theta_b = \frac{K_d \cdot c_{target}}{1 + K_d \cdot c_{target}} \cdot S_{NP} \cdot \theta_a \cdot \theta_b \tag{S6}$$

Given the current response is proportional to the concentration of electrocatalyst (HRP), the current response (i) is:

$$i = k \cdot \frac{n_{cat.}}{S_{electrode}} + constant. = k \cdot \frac{S_{NP}}{S_{electrode}} \cdot \frac{K_d \cdot c_{target}}{1 + K_d \cdot c_{target}} \cdot \theta_a \cdot \theta_b + constant. \tag{S7}$$

From the above equation, we notice that current response (i) depends on nanoparticles size (r_{NP}), bioreceptor-target binding affinity (K_d), bioreceptor (θ_a) and electrocatalyst surface coverage (θ_b), and target concentration (c_{target}). After simplification of the above equation, it was observed that the current-target concentration relationship fits the Langmuir-isotherm

In the case of p53 peptide-HRP system, the total anchoring sites on nanoparticle surface is fixed:

$$\theta_a + \theta_b = \theta_{total} \quad (S8)$$

The voltammetic response (i) is:

$$i = k \cdot \frac{S_{NP}}{S_{electrode}} \cdot \frac{K_d \cdot c_{target}}{1 + K_d \cdot c_{target}} \cdot \theta_a \cdot (\theta_{total} - \theta_a) + constant. \quad (S9)$$

The ratio of p53 peptide to HRP on the particle surface can influence the voltammetric response. As shown in Figure **S19**, a quadratic trend was noted, reflective of the balance between target capture probability and HRP-based signal generation. Therefore, the optimisation of p53 peptide/HRP surface coverage is essential.

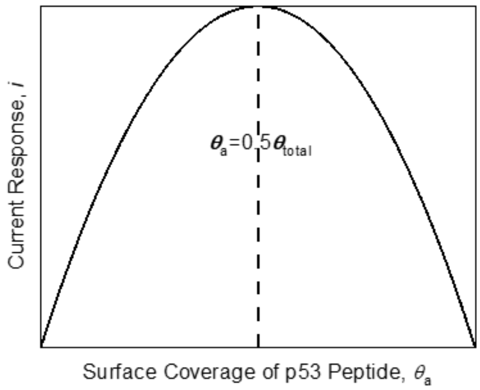

Figure S19. The simulated influence of p53 peptide surface coverage on the voltammetric response. A parabolic curve was found (equation **S9**) with the maximum current response achieved when p53 peptide surface coverage reaches half of the total available binding sites.

REFERENCE

(1) Silva, R. A.; Carmona-Ribeiro, A. M.; Petri, D. F. S. Adsorption behavior and activity of horseradish peroxidase onto polysaccharide-decorated particles. *J. Biol. Macromol.* **2007**, *41* (4), 404-409.

(2) Aibara, S.; YAMASHU, H.; MORI, E.; KATO, M.; MORITA, Y. Isolation and characterization of five neutral isoenzymes of horseradish peroxidase. *J. Biol. Chem.* **1982**, *92* (2), 531-539. Rennke, H. G.; Venkatachalam, M. A. Chemical modification of horseradish peroxidase. Preparation and characterization of tracer enzymes with different isoelectric points. *J. Histochem. Cytochem.* **1979**, *27* (10), 1352-1353.

(3) Chalkias, N. G.; Kahawong, P.; Giannelis, E. P. Activity increase of horseradish peroxidase in the presence of magnetic particles. *J. Am. Chem. Soc.* **2008**, *130* (10), 2910-2911.
